# The association between childhood trauma and adolescent cyberbullying: chain mediating roles of emotional intelligence and online social anxiety

**DOI:** 10.3389/fpsyt.2023.1184382

**Published:** 2023-05-30

**Authors:** Guanghai Cao, Xinyu Wei, Juan Liu, Xianyin Li

**Affiliations:** ^1^College of Education, Qufu Normal University, Qufu, China; ^2^College of Teacher Education, Jining University, Qufu, China

**Keywords:** childhood trauma, emotional intelligence, online social anxiety, cyberbullying, adolescents

## Abstract

**Objective:**

This study explored the impact of childhood trauma on adolescent cyberbullying and the mediating roles of emotional intelligence and online social anxiety between them.

**Methods:**

The Childhood Trauma Scale, Emotional Intelligence Scale, and Chinese Brief Version of the Social Media User Social Anxiety Scale and Cyber Bullying Scale were used to assess 1,046 adolescents [boys: 297, girls: 749, average age = 15.79 years] from four schools in Shandong Province, China. SPSS 25.0 and AMOS 24.0 were used for statistical analysis.

**Results:**

(1) Childhood trauma was positively associated with adolescents’ cyberbullying; (2) Emotional intelligence and online social anxiety played partial mediating roles in the relationship between childhood trauma and cyberbullying; (3) Emotional intelligence and online social anxiety played a chain mediating role in the relationship between childhood trauma and cyberbullying.

**Conclusion:**

This study reveals the relationship and mediating mechanisms between childhood trauma and cyberbullying. It provides implications for the theory and prevention of cyberbullying.

## 1. Introduction

Cyberbullying refers to an assault that an individual or group uses the online media platform to repeatedly and intentionally attack and harm the other individual or group who is unable to protect his/her/themselves (1). In a study on Chinese adolescents, it was found that the incidence of cyberbullying among 5,726 secondary school students over 2 months was 46.8%, of which 19.8% had been bullied, 3.2% had been perpetuating cyberbullying, and 23.8% had been cyberbullied (2). Adolescents are becoming important participants on the Internet owing to online classes. They commit cyberbullying through a variety of channels because of the prevalence and persistence of their Internet use. Due to the anonymous nature of the Internet, adolescents’ behavior on the Internet is more aggressive than that at home and school (3), which may seriously affect the physical and mental health of cyberbullying victims. For example, online bullying can have more negative impacts on the physical, psychological, and academic well-being of victims, and arise more emotional issues related to victimization (4, 5).

Childhood traumatic experiences from the external environment are one of the most important factors in adolescent development (6). Childhood trauma is positively associated with the perpetration of cyberbullying (7), and it is an important determinant of cyberbullying perpetrated by adolescents (8). Studies have shown that childhood trauma and its negative effects are positively associated with pathological use of online applications among adolescents (9), which was, in turn, associated with cyberbullying (10). Concurrently, Sun’s study has suggested that adolescents who experienced more traumatic events during childhood were more likely to relieve negative emotions by engaging in cyberbullying during interpersonal conflicts (11). Many studies demonstrated that childhood trauma was negatively correlated with adolescents’ emotional intelligence, and individuals with low levels of emotional intelligence may experience more negative emotions, such as anger and aggression, than those with high levels of emotional intelligence, and were more likely to perpetrate cyberbullying (12–15). In parallel, it has been shown that negative life events such as childhood trauma affect individuals’ online social anxiety (16, 17), which is also a significant predictor of cyberbullying behavior (18, 19). Most existing research has explored the negative effects of cyberbullying and the relationship between cyberbullying and traditional bullying (20, 21). However, the contributing factors of cyberbullying among adolescents (e.g., childhood trauma and school bullying) have not been well investigated. Therefore, this study explores the roles of two mediating variables—emotional intelligence and online social anxiety—between childhood trauma and adolescent cyberbullying, from the perspective of the general aggression model, thus providing important research support for the reduction and prevention of cyberbullying.

### 1.1. Theoretical framework

Kowalski argued that the general aggression model provided a valuable theoretical framework for explaining cyberbullying (22). The general aggression model assumes that cyberbullying is influenced by personal and situational factors based on knowledge structures (i.e., scripts and schemas). In addition, it assumes that the cognitive, emotional, and arousal pathways may influence current internal states, and the assessment and decision-making processes may determine behavior, contributing to understand the development of cyberbullying through the input processes of personal and situational factors (23). Inputs from personal and situational factors enter the evaluation and decision-making process through their effects on cognition, emotion, and arousal, meanwhile impulsive behaviors in the proximal process develop into cyberbullying, which ultimately leads to negative distal outcomes for adolescents (e.g., psychological health, social functioning, and behavioral problems) (24). According to the general aggression model, cyberbullying is influenced by a combination of individual and environmental factors. Childhood trauma is one of the environmental factors and emotional intelligence and online social anxiety is individual factors, both of them are valid predictor variables of cyberbullying (25–27). The current study used the general aggression model to explain the relationship between childhood trauma, emotional intelligence, online social anxiety, and adolescent cyberbullying.

### 1.2. The relationship between childhood trauma and cyberbullying

Childhood trauma is the abuse and neglect committed by a parent or significant other on children before the age of 16 years. Individuals who are unable to cope appropriately with childhood trauma may experience chronic anxiety and subsequent feelings of hopelessness and helplessness (28, 29). Childhood trauma is a manifestation of life stress and includes emotional, physical, and sexual abuse, and emotional and physical neglect (25). Khine and Turk’s research has found that childhood experiences can lead to negative emotions in adolescents and influence their onset of behavioral problems in the long term (30, 31). The general aggression model identifies the following environmental factors associated with cyberbullying: parental involvement, school climate, and social interaction (22). Childhood trauma caused by parents or occured in the school environment is one such environmental factor that serves as a valid predictor variable for cyberbullying (25, 32). It was found that emotional neglect, emotional abuse, physical abuse, sexual abuse, and somatic neglect were all positively associated with the perpetration of cyberbullying (33–36). Accordingly, this study proposes research Hypothesis 1: *childhood trauma has positive influence on adolescent cyberbullying.*

### 1.3. The mediating role of emotional intelligence

Of the two factors that influence cyberbullying—individual and environmental—individual factors include personal beliefs, attitudes, values, and other stable psychological characteristics. Emotional intelligence is an individual factor and a valid predictor variable of cyberbullying perpetration (22). Emotional intelligence refers to an individual’s ability to reason and use emotional information to guide their thinking and actions, including the ability to accurately assess their own and others’ emotions, express and regulate emotions adaptively, understand emotions and emotional knowledge, and use emotional information to solve problems (37). Emotional intelligence, as the ability to control one’s own emotions and recognize the emotions of others, plays a significant role in adolescents’ development (38). However, empirical research has demonstrated that childhood trauma can negatively impact an individual’s emotional intelligence (39–41). For example, Jehan found that childhood abuse reduced the ability to use one’s own emotions and recognize the emotions of others (42), and thus led to mood disorders (43). Several studies have likewise explored the relationship between childhood trauma and emotional intelligence in different dimensions and found that childhood psychological abuse, psychological neglect, emotional abuse, emotional neglect, and physical abuse all negatively affected emotional intelligence (44–47).

Further, researchers have found that a lack of emotional regulation skills underlies the generation of cyber conflict (26), increasing the probability of aggression. Prior studies (2014) found that emotional intelligence was negatively associated with cyberbullying aggression (13–15). Higher levels of emotional intelligence could be associated with less cyberbullying, and adolescents with lower emotional intelligence scored higher on negative emotions such as aggression, anger, and hostility (12), and exhibited more cyberbullying (26). A study on university students revealed that bullies had lower emotional intelligence than non-bullies, confirming the relationship between emotional intelligence and cyberbullying (48). Bullies may be characterized as lacking emotional skills in life (49–51); being unable to express, understand, or regulate their emotions; and having lower levels of emotional attention, discrimination, and comprehension of others’ emotions (52). This emotional deficit may lead to difficulties in understanding and managing their negative emotions or even identifying them (53). Meanwhile, the adolescent education program developed by Schoeps et al. demonstrated that by training adolescents’ emotional intelligence and guiding them to better recognize and regulate their emotions, the probability of cyberbullying can be reduced and adolescents’ subjective well-being can be enhanced (54). These existing studies supported the view that the characteristics of emotional intelligence make it one of the strongest protective factors against the emergence of cyberbullying (55, 56). Accordingly, this study proposes research Hypothesis 2: *emotional intelligence mediates the relationship between childhood trauma and adolescent cyberbullying.*

### 1.4. The mediating role of online social anxiety

Based on the general aggression model, online social anxiety, as one of the internal states of the individual, is also an influential factor in cyberbullying behavior. Individuals with a history of childhood trauma would experience a variety of negative outcomes during their development, including social anxiety (57). Parental rejection from childhood trauma was found to be associated with social anxiety in a community sample of adolescents (58). As the boundaries between online and offline interactions become increasingly blurred, online media interactions can also trigger social anxiety in individuals and even give rise to a new form of anxiety (59), namely, online social anxiety. Online social anxiety refers to the negative interpersonal experience of tension, anxiety, and fear when individuals use social media to interact with others. It encompasses three aspects: interaction anxiety, privacy concerns, and evaluation fears (60). Online social anxiety is considered a form of state anxiety, which is manifested by the perception of possible danger in virtual spaces (61). Some studies have shown that negative life events are significantly and positively correlated with state anxiety, in other words, negative life events could affect individuals’ levels of online social anxiety (16, 17).

Chinese scholars have demonstrated that the effects of early negative life events, such as childhood traumatic experiences, would continue into adulthood, hence, maltreated individuals may continue to experience high levels of online social anxiety in social interactions, even after they remove from the previous life circumstances (62–67). In contrast, adolescents under the age of 16 years are temporarily unable to break away from their previous life circumstances and often remember these negative experiences when faced with such circumstances, producing high levels of social anxiety (57). A significantly positive correlation between online social anxiety and cyberbullying has been demonstrated (27). Although online social anxiety has been identified as a significant predictor of cyberbullying, few studies have investigated its longitudinal association with cyberbullying (19, 68). Some studies demonstrated that high levels of online social anxiety were prevalent among cyberbullies (19); as the level of online social anxiety increases, the likelihood of aggressive behavior also increases (69). Stable cyberbullying perpetrators have demonstrated higher levels of anxiety than other students (70). This further suggests that anxiety symptoms are a risk factor for cyberbullying. Moreover, anxiety has been suggested as a significant predictor of cyberbullying behavior (18, 19). Therefore, we propose Hypothesis 3: *childhood trauma indirectly influences adolescent cyberbullying behavior through online social anxiety*.

### 1.5. The chain mediating role of emotional intelligence and online social anxiety

Current research has indicated that adolescent online social anxiety is significantly and negatively related to emotional intelligence (71), implying that emotional intelligence is a significantly negative predictor of social anxiety and increasing emotional intelligence can reduce online social anxiety (72). A significant negative correlation between emotional intelligence and online social anxiety was explored and verified in a non-clinical context (71). Likewise, emotional intelligence was demonstrated to indirectly affect social anxiety by influencing interpersonal adaptation (72). In addition, individuals with high emotional intelligence had higher online interpersonal perceptions and lower online social anxiety when they perceived the dangers of online communities (61). The level of psychological stress that individuals experienced when integrating into different online social groups due to incompatible expectations and demands could further increase individuals’ online social anxiety levels (59). Research has shown that emotional intelligence mediates the relationship between gray matter volume in middle temporal gyrus and social anxiety among late adolescents (73). In addition, earlier research found that emotional intelligence explained additional differences in online social anxiety even after adjusting for variables such as anxiety, self-esteem, weight, overall psychological functioning, and demographic characteristics (74–76). Adolescents’ emotional intelligence is strongly related to online social anxiety, and increasing emotional intelligence may be an effective way to reduce anxiety. Adolescents who suffered from traumatic childhood experiences had a reduced ability to control their emotions and identify the emotions of others, negatively impacting their emotional intelligence (42). Individuals with lower emotional intelligence could feel uncomfortable in online social interactions due to interpersonal stress, further increasing their levels of online social anxiety (60). As the level of online social anxiety increases, the likelihood of individuals engaging in aggressive behavior also increases (69). Accordingly, we propose Hypothesis 4: *childhood trauma impacts adolescent cyberbullying through a chain mediating effect of emotional intelligence and online social anxiety*.

### 1.6. Current research

**Figure 1:**
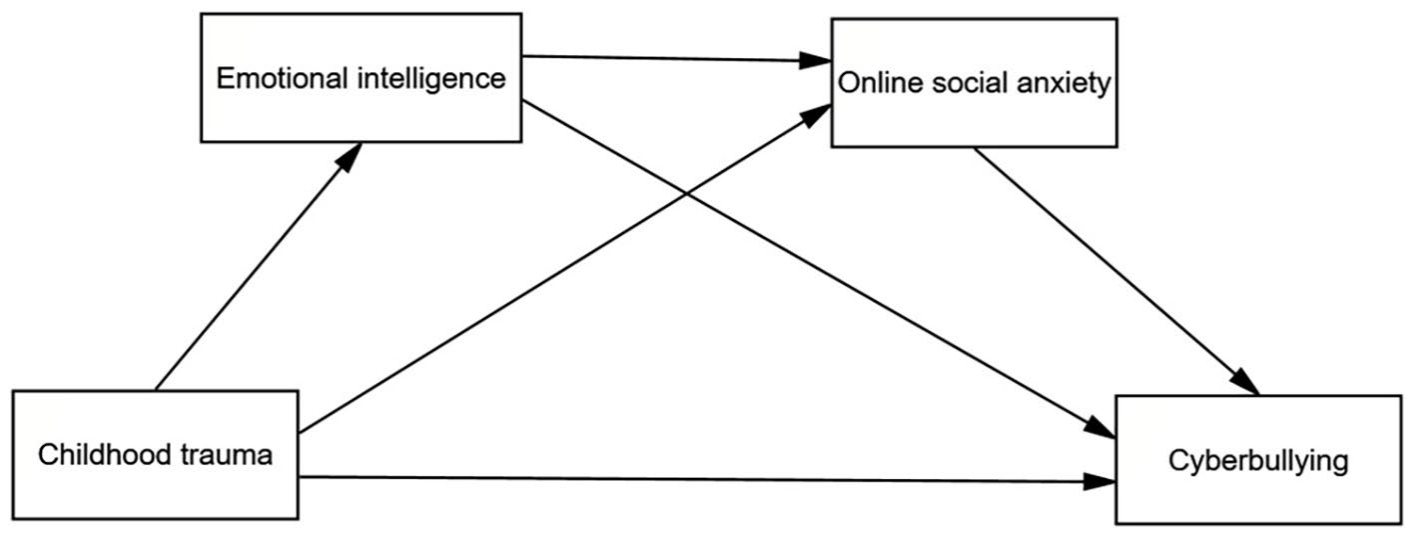
Hypothetical model for mediating emotional intelligence and online social anxiety between childhood trauma and cyberbullying.

This study examined the mediating roles of emotional intelligence and online social anxiety in the relationship between childhood trauma and cyberbullying using a sample of Chinese adolescents. Figure 1 shows the hypothetical model of the study. By examining the influence of adolescents’ childhood trauma on cyberbullying and the mediating roles of emotional intelligence and online social anxiety, this study expands the current understanding of the mediating mechanism related to cyberbullying. A better understanding provides a strong theoretical basis for effective intervention and reduction of cyberbullying among adolescents.

## 2. Methods

### 2.1. Participants and procedures

The survey was conducted using an online web-based questionnaire, and 1,245 students from secondary schools across China were randomly surveyed. The questionnaire comprised 80 items. The survey was conducted with reference to the completion time of 60 respondents in the pre-survey, and those samples with a completion time of less than 180 s in the official questionnaire were judged invalid. The final number of valid questionnaires was 1,046, with 297 (28.39%) boys and 749 (71.61%) girls. The participants’ average age was 15.78 years.

This study was approved by the ethics committee of the author’s affiliated institution. All students were informed of the study purpose and that the results would be used for research. Written informed consent was obtained from both teachers and parents, and all participants provided verbal informed consent. Subsequently, the students completed the questionnaires in their computer classrooms, guided by trained researchers. The questionnaire included demographic information, scales of childhood trauma, emotional intelligence, online social anxiety, and cyberbullying.

### 2.2. Measures

#### 2.2.1. Childhood trauma scale

Childhood trauma was measured using the Childhood Trauma Scale developed by Bernstein et al. in 1998 (77). The 28-item scale comprises five factors: emotional abuse (EA) (e.g., “People in my family call me ‘stupid’ or ‘lazy’ or ‘ugly’.”), physical abuse (PA) (e.g., “Someone in my family beat me up so badly I had to go to the hospital.”), sexual abuse (SA) (e.g., “Someone has tried to touch me or get me to touch him in a sexual way”), emotional neglect (EN) (e.g., “Someone in my family makes me feel important or special”), and physical neglect (PN) (e.g., “I cannot get enough to eat.”). The questionnaire is based on a five-point Likert scale (1 = “*never*” and 5 = “*always*”). Questions 10, 16, and 22 of the scale were denial validity scales, which were used to detect underreporting of trauma and were therefore not scored. The total score of the Childhood Trauma Scale ranged from 25 to 125, with higher scores indicating more severe childhood traumatic experiences. Scores of PN ≥ 10, EN ≥ 15, SA ≥ 8, PA ≥ 10, and EA ≥ 13 are considered to indicate having undergone traumatic experiences in childhood (78). The Chinese version of the Childhood Trauma Scale used in this study was translated by Zhao Hao et al. (79). The internal consistency coefficient and construct validity of the Childhood Trauma Scale in this study was 0.623 and *χ^2^/df(A statistical measure for directly testing the similarity between the sample covariance matrix and the estimated variance matrix)* = 4.877, GFI(goodness-of-fit index) = 0.909, CFI(comparative fit index) = 0.883, and RMSEA(root-mean-square error of approximation) = 0.061. These indicate that this scale has good reliability and validity.

#### 2.2.2. Emotional intelligence scale

The Emotional Intelligence Scale, translated by Wang and Law in 2004 (80), was used to assess individuals’ levels of emotional intelligence. It comprises 16 items in four dimensions: emotional assessment of self (e.g., “I am a self-motivated person.”), emotional assessment of others (e.g., “I can always tell my friends’ emotions from their actions.”), emotion management (e.g., “I have good control over my emotions.”), and emotional use (e.g., “I always set goals for myself and try my best to accomplish them.”). The questionnaire is based on a seven-point Likert scale (1 = “*strongly disagree*” and 7 = “*strongly agree*”). The total score is the sum of the scores of each question and ranges from 16 to 112. The higher the score, the higher the level of emotional intelligence. Cronbach’s alpha for this scale in this study was 0.959 and the fit indicators were *χ*^2^*/df* = 5.581, GFI = 0.952, CFI = 0.977, and RMSEA = 0.066. These indicate that this scale has good reliability and validity.

#### 2.2.3. Online social anxiety scale

Online Social anxiety was measured using the Chinese version of the Social Media User Anxiety Inventory (81), revised by Chen et al. It comprises 20 items in three dimensions: appraisal fear (e.g., “On social media, I worried that people would find it embarrassing.”), privacy concerns (e.g., “When using social media, I often feel uneasy about the possibility of my personal information being made public.”), and interaction anxiety (e.g., “I feel uncomfortable talking to people I’ve just met on social media.”). The questionnaire is based on a five-point Likert scale (1 = “*not at all*” and 5 = “*completely*”). The total score of the scale was summed across all items, and ranged from 20 to 100, with higher scores indicating higher levels of online social anxiety. Cronbach’s alpha of the scale in this study was 0.969, and the fit indicators were *χ*^2^*/df* = 5.648, GFI = 0.919, CFI = 0.966, and RMSEA = 0.067. These indicate that this scale has good reliability and validity.

#### 2.2.4. Cyberbullying scale

Cyberbullying was measured using the Implementing Cyberbullying Behavior Scale from the Chinese version of the Cyberbullying Scale revised by Youyang (82). This scale measures the frequency of cyberbullying in the preceding 1 year and comprises eight items in three dimensions: cyber verbal bullying (e.g., “When I encounter someone scolding me online, I will also scold them.”), anonymity (A) (e.g., “If you see software that allows you to spy on others, you will want to use it and download it.”), and cyber fake bullying (e.g., “If I get a dirty picture, I’ll find a way to get a dirty picture of others.”). The questionnaire uses a five-point Likert scale (1 = “*never happens*” and 5 = “*always happens*”). The total score of the scale was summed across all questions and ranged from 8 to 40, with higher scores indicating higher levels of involvement in cyberbullying. Cronbach’s alpha for the scale in this study was 0.848, and the fit indicators were *χ*^2^*/df* = 3.734, GFI = 0.991, CFI = 0.993, and RMSEA = 0.051. These indicate that this scale has good reliability and validity.

### 2.3. Data analysis

Data were analyzed using SPSS 25.0 and Amos 24.0. First, descriptive statistics and correlation analysis were conducted. Second, structural equation model analysis and bias-corrected percentile Bootstrap method (5,000 repetitions) using the maximum likelihood estimation method of Amos 24.0 statistical software were conducted to evaluate structural models to test for mediating effects (83). Two types of indices were used for the goodness-of-fit: relative and absolute goodness-off-fit indices. The former included the CFI, Tucker-Lewis Coefficient (TLI), and incremental fit index (IFI). The latter comprised *χ*^2^/df, the RMSEA, standardized root mean square residual (SRMR), GFI, and adjusted goodness of fit index (AGFI).

## 3. Results

### 3.1. Common method deviation control and testing

The data for this study were all derived from questionnaires, which may be subject to common method bias. According to previous recommendations, a Harman one-way test for common method bias was used (84). The results showed that a total of 13 factors with characteristic roots greater than 1 were extracted from the unrotated factor analysis results, of which the first factor explained 22.209% of the variance, which was below the critical criterion of 40%. This indicated that there was no significant common method bias in this study.

### 3.2. Descriptive analysis of the variables and their correlation analysis

Table 1 shows that childhood trauma was significantly and positively correlated with cyberbullying and online social anxiety, while significantly and negatively correlated with emotional intelligence; cyberbullying was significantly and positively correlated with online social anxiety, while significantly and negatively correlated with emotional intelligence; emotional intelligence was significantly and negatively correlated with online social anxiety.

**Table 1:** Descriptive statistics for each scale.

Variables	*M* ± SD	Childhood trauma	Emotional intelligence	Online social anxiety	Cyberbullying
Childhood trauma	33.29 ± 7.32	1			
Emotional intelligence	85.82 ± 17.10	−0.375**	1		
Online social anxiety	53.61 ± 20.01	0.165**	−0.246**	1	
Cyberbullying	11.36 ± 4.70	0.225**	−0.217**	0.227**	1

**p < 0.01.

### 3.3. Correlation coefficients between each dimension of childhood trauma and the three variables

Table 2 shows that each dimension of childhood trauma was significantly correlated with emotional intelligence, online social anxiety, and cyberbullying, among which emotional abuse has the highest correlation with these three variables.

**Table 2:** Correlation coefficients of each dimension of childhood trauma with each variable.

Variables	Childhood trauma	Emotional intelligence	Online social anxiety	Cyberbullying
Emotional abuse	0.732**	−0.257**	0.215**	0.274**
Physical abuse	0.571**	−0.143*	0.086**	0.134**
Sexual abuse	0.436**	−0.144**	0.089**	0.152**
Emotional neglect	0.808**	−0.324**	0.064**	0.111**
Somatic neglect	0.693**	−0.286**	0.127**	0.137**

*p < 0.05; **p < 0.01.

### 3.4. Structural model

A hypothesis model was constructed by sorting out childhood trauma, emotional intelligence, online social anxiety, and adolescent cyberbullying to obtain a mediation model with the childhood trauma as independent variables and emotional intelligence and online social anxiety as mediating variables that together acted on adolescent cyberbullying. Table 3 shows that the structural model fits well with the fitted values of *χ*^2^/df = 5.190, GFI = 0.945, CFI = 0.954, TLI = 0.943, IFI = 0.955, AGFI = 0.921, SRMR = 0.032, and RMSEA = 0.065.

**Table 3:** Goodness-of-fit indices for structural models.

Fit index	*χ*^2^/df	SRMR	RMSEA	GFI	AGFI	IFI	CFI	TLI
Recommended value	0–5	<0.080	0–0.080	>0.900	>0.900	>0.900	>0.900	>0.900
Values for this study	5.19	0.032	0.063	0.945	0.921	0.955	0.954	0.943

### 3.5. The mediating role of emotional intelligence and online social anxiety in the relationship between childhood trauma and cyberbullying

**Figure 2:**
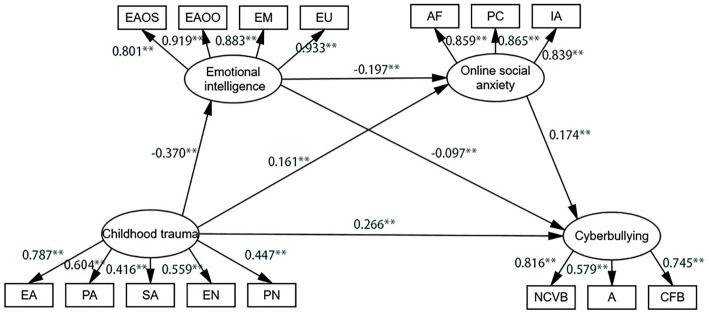
Multiple mediation model. Path values are the path coefficients (Standardization coefficient). **p < 0.01.

As shown in Figure 2, the model indicated that childhood trauma was significantly associated with online social anxiety and cyberbullying (*β* = 0.161, *p* < 0.01; *β* = 0.266, *p* < 0.01) and significantly negative associated with emotional intelligence (*β* = −0.370, *p* < 0.01). Furthermore, emotional intelligence (*β* = −0.197, *p* < 0.01; *β* = −0.097, *p* < 0.01) and online social anxiety (*β* = 0.174, *p* < 0.01) were significantly negative and positive correlated with cyberbullying, respectively.

As shown in Table 4, the total effect of childhood trauma on adolescent cyberbullying was 0.791 and the direct effect was 0.614. Emotional intelligence and online social anxiety partially mediated the relationship between childhood trauma and cyberbullying, with a mediating effect of 0.177, accounting for 22.38% of the total effect. The mediating effect comprised three pathways, namely, indirect pathway 1: childhood trauma → emotional intelligence → cyberbullying (effect value was 0.083); indirect pathway 2: childhood trauma → online social anxiety → cyberbullying (effect value was 0.065); and indirect pathway 3: childhood trauma → emotional intelligence → online social anxiety → cyberbullying (effect value was 0.029). The effect values for these three pathways accounted for 10.49, 8.22, and 3.37% of the total effect, respectively, and the Bootstrap 95% confidence intervals for all three pathways did not contain 0, indicating that all three mediating effects reached significant levels.

**Table 4:** Bootstrap analysis of the mediating effects test.

	Paths	Effect value	Boot SE	BootLLCI	BootULCI
Direct effect	Childhood Trauma → Cyberbullying	0.614	0.158	0.334	0.953
Indirect effects	Ind1	0.083	0.04	0.01	0.167
	Ind2	0.065	0.022	0.031	0.117
	Ind3	0.029	0.009	0.015	0.051
Total effect	Childhood Trauma → Cyberbullying	0.791	0.155	0.518	1.129

Ind1: Childhood trauma → emotional intelligence → cyberbullying.

Ind2: Childhood trauma → online social anxiety → cyberbullying.

Ind3: Childhood trauma → emotional intelligence → online emotional intelligence → cyberbullying.

## 4. Discussion

This study aimed to explore the influence of childhood trauma on adolescent cyberbullying and examine the chain mediating role of individual factors (emotional intelligence and online social anxiety) in this relationship from the perspective of the general aggression model. The study revealed the generation and development of cyberbullying among adolescents in a more comprehensive way, and the results of the study can provide a reference for empirical studies of cyberbullying worldwide. Therefore, it is beneficial to explain the causes of cyberbullying in the era of big data and provide a new perspective for intervening in adolescent cyberbullying and promote the healthy physical and psychological development of adolescents.

### 4.1. The relationship between childhood trauma and cyberbullying

The study demonstrated a significant positive association between childhood trauma and cyberbullying, confirming Hypothesis 1. It also found that all dimensions of childhood trauma were positively associated with cyberbullying, again confirming that childhood trauma is a significant predictor of cyberbullying (33–36). Childhood trauma is the ultimate source of the “cycle of violence” (85), and children who have experienced trauma are more likely to perpetrate violence. Adolescents who are traumatized in their family are more likely to engage in aggressive behavior than those who are not traumatized (86) for two reasons. First, adolescents who have experienced childhood trauma often grow up feeling fearful, angry, lonely, rejected, denied, and afraid of failure, as well as having many uncertainties and not knowing appropriate ways to express their negative emotions. These would increase their likelihood of perpetrating cyberbullying (33). Second, adolescents who have experienced childhood trauma tend to isolate themselves, perceive everyone as insecure, and have low self-trust, self-esteem, and sense of value, but have high expectations of themselves. This contradiction makes them vulnerable to high frequency of aggressive behaviors (4), and their aggressive behaviors are more often manifested through cyberbullying. Therefore, adolescents with more severe childhood trauma are more likely to commit cyberbullying.

### 4.2. Analysis of chain mediating effects of emotional intelligence and online social anxiety

A chain mediation model was developed to account for the relationship between childhood trauma and cyberbullying and to elaborate on the mechanisms by which emotional intelligence and online social anxiety play mediating roles between the two.

First, the study showed that emotional intelligence mediated the relationship between childhood trauma and adolescent cyberbullying, which confirmed Hypothesis 2. The present study showed that childhood trauma was negatively associated with emotional intelligence. This is consistent with previous findings that adolescents with higher levels of childhood trauma have lower emotional intelligence (39, 40). Adolescents who have experienced abuse or trauma are emotionally controlled by others, and they tend to express their emotions passively and negatively, communicate poorly, and easily interpret the emotions of others as potential danger signals and become hostile to others (87), Traumatic childhood experiences can inhibit individuals from learning how to properly use and understand emotional information from life events, while these negative life events can lead to a reduction in the volume of the corpus callosum in the brain dedicated to a range of higher cognitive, emotional, and other information transfer functions, further impairing the development of emotional competence (88). Simultaneously, the current research showed that emotional intelligence was negatively associated with cyberbullying, with individuals of lower emotional intelligence exhibiting more aggressive behavior, which was also consistent with previous research (13, 15). The more traumatized an individual is in childhood, the worse their emotional experience and expression will be, and the more problems they will have with emotional communication and interpersonal interactions in their lives, resulting in lower emotional intelligence (89). Adolescents with low emotional intelligence are unable to regulate their emotions and recognize the emotions of others. They tend to be rejected or discouraged by others in interpersonal interactions, thus causing them to have more negative emotions and increasing their likelihood of cyberbullying. In contrast, adolescents with higher emotional intelligence have higher levels of emotional understanding, and emotional regulation, and empathy (90), and they have fewer negative emotions (15, 91), reducing their involvement in cyberbullying. Thus, cyberbullying can be considered an aggressive response to negative emotions triggered by low emotional intelligence caused by childhood trauma.

Second, the study showed that online social anxiety played a mediating role in the relationship between childhood trauma and adolescent cyberbullying, verifying Hypothesis 3. Childhood trauma was significantly and positively associated with online social anxiety, which was consistent with previous studies (57). Childhood trauma can negatively affect individuals’ mental health, such as triggering negative emotions (e.g., sadness, shame, and fear), leading to their withdrawn personalities and dislike of interacting with others, causing them to show more avoidance and withdrawal behaviors in later interpersonal interactions, increasing the risk of online social anxiety. Simultaneously, online social anxiety was positively associated with adolescent cyberbullying, and as the level of online social anxiety increases, the likelihood of aggressive behavior among individuals increases as well (69). It can exacerbate their psychological and interpersonal stress. Furthermore, the more severe the rejection and disregard from peers, the more severe the anxiety and irritability generated when interacting with people. These negative emotional experiences can exacerbate the aggressiveness of individuals, who, owing to the anonymity and unrestricted nature of the Internet, have the potential to increase their connectivity through social media, thus increasing the likelihood that they will perpetrate cyberbullying (92).

Finally, the study revealed the chain mediation of emotional intelligence and online social anxiety in the relationship between childhood trauma and adolescent cyberbullying, verifying Hypothesis 4. Individuals with high emotional intelligence are able to detect others’ emotions in a timely and accurate manner in their daily social activities, form more positive interactions with others, and effectively deal with stress from social interactions. Individuals with low emotional intelligence are unable to develop good interpersonal interactions and online interpersonal perceptions (72), resulting in high levels of online social anxiety, further increasing the occurrence of cyberbullying. Moreover, adolescents with high emotional intelligence are able to reasonably assess their emotions when facing stress and difficulties in life, find appropriate ways to adjust when negative emotions are formed, and reduce their negative emotions (93).

### 4.3. Implications and limitations

This study explored the fundamental causes and mechanisms of adolescent cyberbullying based on the general aggression model, providing an important theoretical basis for preventing and reducing adolescent cyberbullying. This study explored the internal mechanisms by which childhood trauma influenced cyberbullying and identified three important pathways of action: childhood trauma → emotional intelligence → cyberbullying; childhood trauma → online social anxiety → cyberbullying; and childhood trauma → emotional intelligence → online social anxiety → cyberbullying. These results theoretically extend the general aggression model to explore cyberbullying behaviors and influencing factors in adolescent groups, not only explaining the influence of childhood trauma on cyberbullying, but also the mechanism of action by which this influence arises. Additionally, these findings provide insight into the prevention of cyberbullying, enrich the existing research literature, and provide implications for future research. Practically, this study suggests that cyberbullying can be indirectly prevented and controlled by increasing emotional intelligence and reducing the online social anxiety level of individuals who have experienced childhood trauma. As an early negative life event, childhood trauma can considerably damage children’s physical and mental health, and its negative effects may not subside for decades. Accordingly, this should be taken seriously by families, schools, and society. In the process of adolescents’ growth, parents should pay attention to their daily lives, encourage adolescents to express their emotions correctly, and consciously guide adolescents to feel emotional changes and express their emotional experiences in a timely manner through language and writing. Schools should actively conduct programs to improve students’ emotional intelligence and reduce their online social anxiety, guide adolescents to better understand and regulate their emotions, establish a sense of proper emotion management, reduce their online social anxiety level, and train good online social skills to improve their interpersonal relationships, improve adolescents’ mental health, and further reduce and avoid the occurrence of cyberbullying. At present, domestic and international research on the relationship between childhood trauma and youth cyberbullying is incomplete, Most existing research explores the negative effects of cyberbullying and the relationship between cyberbullying and traditional bullying (20, 21). But the underlying causes of the occurrence of cyberbullying in adolescents (e.g., the effects of childhood trauma such as parental abuse and bullying in school) are not well researched. And the pathways of how childhood trauma influences cyberbullying need to be further explored in future empirical studies.

This study has some limitations. First, it is a cross-sectional study with varying degrees of recall bias, which cannot fully explain the causal relationship between childhood trauma, emotional intelligence, online social anxiety, and cyberbullying. Future research should adopt a longitudinal approach, which would allow researchers to better assess the cyberbullying behavior of participants who have suffered traumatic childhood experiences, in addition to intervening and ameliorating cyberbullying behaviors caused by such trauma. Second, the data for each variable were obtained from participants’ self-reports. As such, participants may have experienced social approval effect, leading to questionnaire responses that do not fully and accurately reflect their true situations. To further improve the results and validity of this study, we recommend that future studies use objective measurement tools or add other sources of information to assess these variables. Similarly, the CR (Construct Reliability) and AVE (Average Variance Extracted) data in this study’s indicators are not satisfactory and should be improved in future studies. Finally, the study sample only included Chinese secondary school students, whose academic interests inclined to the liberal arts, with more girls than boys; consequently, the findings may not generalize to other cultural contexts. However, as the variables assessed in this study may be expected to show similar relationships in other populations, future studies should expand the scope to include other cultural contexts.

## 5. Conclusion

The findings of this study can be summarized as follows. (1) Childhood trauma, emotional intelligence, and online social anxiety are significantly correlated with each other; childhood trauma and online social anxiety are significantly positively correlated with cyberbullying and emotional intelligence are significantly negatively correlated with cyberbullying. (2) Childhood trauma influences cyberbullying indirectly through emotional intelligence and online social anxiety. (3) Childhood trauma can affect cyberbullying directly or through the “emotional intelligence – online social anxiety” mediating chain.

## Data availability statement

The original contributions presented in the study are included in the article/supplementary material, further inquiries can be directed to the corresponding author.

## Ethics statement

The studies involving human participants were reviewed and approved by the Biomedical Ethics Committee of Qufu Normal University. Written informed consent to participate in this study was provided by the participants’ legal guardian/next of kin. Written informed consent was obtained from the individual(s) for the publication of any potentially identifiable images or data included in this article.

## Author contributions

GC: methodology, validation, investigation, resources, data management, written review and editing, project management, funding acquisition, and supervision. XW: conceptualization, methodologies, software, investigation, writing, and editing. JL: software, writing, revision, and editing. XL: formal analysis, written review, editing, and supervision. All authors in this study contributed to the article and approved the submitted version.

## Conflict of interest

The authors declare that the research was conducted in the absence of any commercial or financial relationships that could be construed as a potential conflict of interest.

## Publisher’s note

All claims expressed in this article are solely those of the authors and do not necessarily represent those of their affiliated organizations, or those of the publisher, the editors and the reviewers. Any product that may be evaluated in this article, or claim that may be made by its manufacturer, is not guaranteed or endorsed by the publisher.
